# Methylation Status of Exon IV of the Brain-Derived Neurotrophic Factor (BDNF)-Encoding Gene in Patients with Non-Diabetic Hyperglycaemia (NDH) before and after a Lifestyle Intervention

**DOI:** 10.3390/epigenomes6010007

**Published:** 2022-02-18

**Authors:** Helene A. Fachim, Nagaraj Malipatil, Kirk Siddals, Rachelle Donn, Gabriela Y. Cortés, Caroline F. Dalton, J. Martin Gibson, Adrian H. Heald

**Affiliations:** 1 The School of Medicine and Manchester Academic Health Sciences Centre, University of Manchester, Manchester M13 9PL, UK; nsmalipatil@gmail.com (N.M.); kirk.siddals@manchester.ac.uk (K.S.); rachelle.donn@manchester.ac.uk (R.D.); martin.gibson@manchester.ac.uk (J.M.G.); 2 Department of Diabetes and Endocrinology, Salford Royal Hospital, Salford M6 8HD, UK; 3 National Research Coordination, Subdirección de Servicios de Salud, Petróleos Mexicanos, Mexico City 11320, Mexico; medic_gaby@live.com.mx; 4 Biomolecular Research Centre, Sheffield Hallam University, Sheffield S1 1WB, UK; c.f.dalton@shu.ac.uk

**Keywords:** BDNF, non-diabetic hyperglycaemia, NDH, T2DM, lifestyle change

## Abstract

BDNF signalling in hypothalamic neuronal circuits is thought to regulate mammalian food intake. In light of this, we investigated how a lifestyle intervention influenced serum levels and DNA methylation of BDNF gene in fat tissue and buffy coat of NDH individuals. In total, 20 participants underwent anthropometric measurements/fasting blood tests and adipose tissue biopsy pre-/post-lifestyle (6 months) intervention. DNA was extracted from adipose tissue and buffy coat, bisulphite converted, and pyrosequencing was used to determine methylation levels in exon IV of the BDNF gene. RNA was extracted from buffy coat for gene expression analysis and serum BDNF levels were measured by ELISA. No differences were found in BDNF serum levels, but buffy coat mean BDNF gene methylation decreased post-intervention. There were correlations between BDNF serum levels and/or methylation and cardiometabolic markers. (i) Pre-intervention: for BDNF methylation, we found positive correlations between mean methylation in fat tissue and waist-hip ratio, and negative correlations between mean methylation in buffy coat and weight. (ii) Post-intervention: we found correlations between BDNF mean methylation in buffy coat and HbA1c, BDNF methylation in buffy coat and circulating IGFBP-2, and BDNF serum and insulin. Higher BDNF % methylation levels are known to reduce BNDF expression. The fall in buffy coat mean BDNF methylation plus the association between lower BDNF methylation (so potentially higher BDNF) and higher HbA1c and serum IGFBP-2 (as a marker of insulin sensitivity) and between lower serum BDNF and higher circulating insulin are evidence for the degree of BDNF gene methylation being implicated in insulinisation and glucose homeostasis, particularly after lifestyle change in NDH individuals.

## 1. Introduction

Brain-derived neurotrophic factor (BDNF) is a member of the neurotrophin family of growth factors that promote neuronal differentiation, adult neurogenesis, learning, memory [1,2] and neural plasticity [3,4]; it is produced and released by the brain into circulation. In addition to these roles, BDNF has also been identified as a key component of the hypothalamic pathway that controls body weight, food intake, and energy homeostasis in mammals [5].

There is substantial evidence linking BDNF to the progression of type 2 diabetes mellitus (T2DM). Plasma BDNF levels are decreased in T2DM compared to healthy controls and is thought to play a role in systemic inflammation and to interact with several inflammatory cytokines [4]. BDNF is widely expressed throughout the hippocampus and other brain areas as well as peripheral tissues important in the regulation of energy homeostasis, such as adipose tissue, skeletal and smooth muscle, and liver [6,7]. Furthermore, BDNF treatment in obese and diabetic rodents significantly reduces blood glucose level, attenuates body weight gain and food intake, and enhances energy and glucose metabolism [8,9].

Humans with mutations in the BDNF gene [10] or in the trkB receptor signal transduction pathway [11] exhibit severe obesity. Additionally, *BDNF* gene variants are associated with higher risk for increased body weight in both children and adults [12,13]. Similar findings have been shown in mice where reduced *BDNF* expression, either due to a brain specific conditional knockout or due to heterozygous gene expression, leads to hyperphagia, obesity, and insulin and leptin resistance [14,15].

Non-diabetic hyperglycaemia (NDH) refers to a metabolic state between normal glucose homeostasis and diabetes [16]. Additionally, referred to as pre-diabetes or impaired glucose regulation (IGR), NDH represents a high-risk state for the future development of T2DM. T2DM is thought to arise from a summation of genetic and environmental/epigenetic factors, which result in a decline in insulin activity, often on the background of longstanding elevation of body mass index (BMI) with attendant insulin resistance.

Biomarkers are necessary to better assess the trajectory towards T2DM, to monitor responses to treatment, and to personalise therapy, with the aim being to develop new strategies to prevent the progress from NDH to T2DM. BDNF is a potential biomarker. However, studies regarding DNA methylation alteration in key CpGs of the regulatory *BDNF* region in T2DM and obesity are still scarce and warrant further investigation.

In this study, we sought to determine whether a lifestyle intervention could influence DNA methylation of *BDNF* and circulating levels of BDNF in individuals with NDH. Additionally, we tested for possible correlations between BDNF methylation and/or serum levels and clinical variables, as BDNF is thought to play an important role in glucose metabolism.

## 2. Results

The clinical and demographic variables for all the participants were described previously [17,18]. In summary, the Care Call intervention resulted in weight reduction and BMI change (*p* < 0.05) but no other clinically significant alterations (i.e., insulin levels, total cholesterol, HbA1C, HOMA-S, HOMA-B, triglycerides).

**Figure 1:**
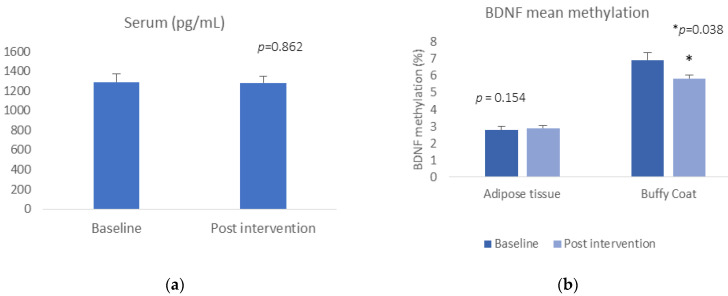
(**a**) BDNF concentration pre- and post-intervention; (**b**) mean BDNF DNA methylation pre and post intervention.

BDNF serum levels or *BDNF* gene expression did not change pre- and post-Care Call intervention (t = −0.177, *p* = 0.862, for serum; t = 1.500, *p* = 0.154 for buffy coat gene expression) (Figure 1a). Regarding the DNA methylation data, we did find a reduction in mean *BDNF* methylation in buffy coat, (buffy coat: t = 2.306, *p* = 0.038) (Figure 1b). There was no significant alteration in methylation at any of the CpG sites or in mean methylation in adipose tissue between baseline and 6-month follow-up after the Care Call intervention (adipose tissue: t = −0.792, *p* = 0.438).

We then tested for Pearson’s correlation the *BDNF* methylation data, buffy coat gene expression, BDNF serum levels and clinical variables at baseline (pre-intervention) and post-intervention (see below).

**Figure 2:**
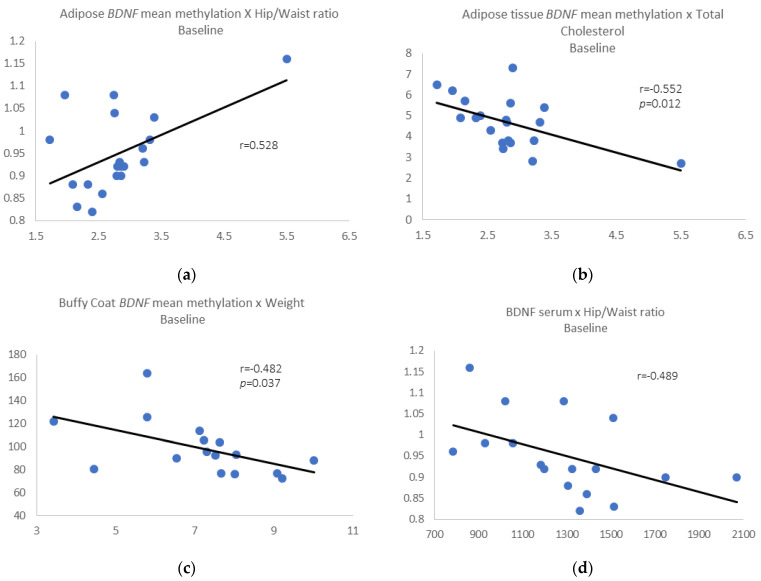
(**a**) Adipose tissue *BDNF* mean methylation vs. waist/hip ratio; (**b**) adipose tissue *BDNF* mean methylation vs. total cholesterol; (**c**) buffy coat *BDNF* mean methylation vs. weight; (**d**) removing those individuals who gain 3% or more in weight, BDNF serum levels vs. waist/hip ratio.

Pre-intervention results: We identified a positive correlation between *BDNF* mean methylation in adipose tissue and waist/hip ratio (r = 0.528, *p* = 0.017) (Figure 2a) and negative correlations between adipose *BDNF* mean methylation and total cholesterol (r = −0.552, *p* = 0.012) (Figure 2b).

We also found a negative correlation between BDNF mean methylation in buffy coat and weight (r = −0.482, *p* = 0.037) (Figure 2c). When we removed those individuals who gain 3% or more in weight, all the correlations described above were maintained, and we also found a negative correlation between BDNF serum levels and waist/hip ratio (r = −0.489, *p* = 0.046) (Figure 2d).

**Figure 3:**
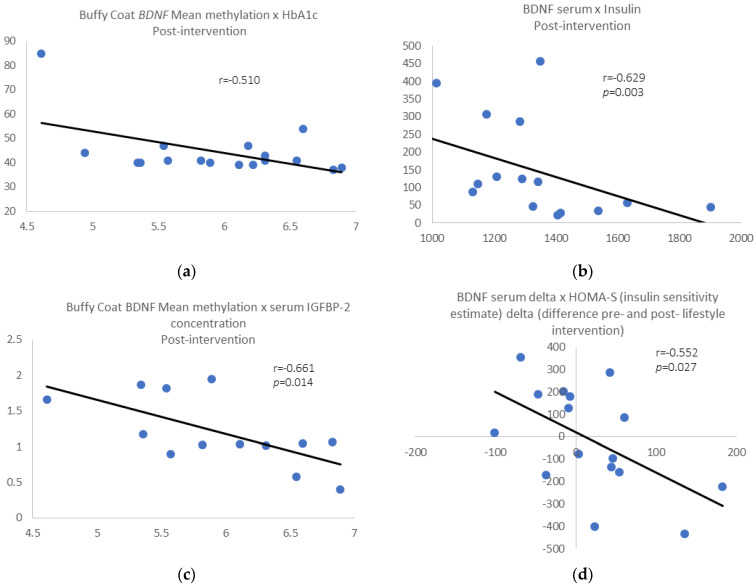
(**a**) Buffy wCoat *BDNF* mean methylation vs. HbA1c; (**b**) serum BDNF levels vs. plasma insulin; (**c**) *BDNF* mean methylation vs. serum insulin-like growth factor binding protein-2 (IGFBP-2) levels. (**d**) BDNF serum delta pre- and post-intervention vs. HOMA-S delta pre- and post-intervention.

Post-intervention results: We found a negative correlation between buffy coat *BDNF* mean methylation and HbA1c levels (r = −0.510, *p* = 0.036; Figure 3a) and between serum BDNF levels and insulin (r = −0.629, *p* = 0.003; Figure 3b) for the whole group. Removing those individuals who gain 3% of weight or more, the above correlations were still significant, and we found a negative correlation between buffy coat *BDNF* mean methylation and serum insulin-like growth factor binding protein-2 gene (IGFBP-2) levels (r = −0.661, *p* = 0.014; Figure 3c). Additionally, we found a negative correlation between BDNF serum delta pre- and post-intervention and HOMA-S delta (r = −0.552, *p* = 0.027; Figure 3d), pre- and post-intervention.

## 3. Discussion

In the present study, we analysed BDNF serum levels and *BDNF* DNA methylation (adipose tissue and buffy coat) in NDH individuals and the effects in these variables before and after a lifestyle intervention. We also tested these variables for correlations with clinical cardiometabolic data. We saw differences in buffy coat mean *BDNF* methylation but not BDNF serum levels after the intervention. We also potentially found clinically relevant correlations between *BDNF* DNA methylation and weight, total cholesterol, HbA1C levels, and waist/hip ratio, as well as between BDNF serum levels and insulin. The BDNF serum delta was negatively correlated with HOMA-S delta in relation to changes with the lifestyle intervention, revealing an important association between BDNF and insulin sensitivity in our sample.

These observed associations between *BDNF* DNA methylation patterns suggest that BDNF may be involved in the pathophysiological process of insulin resistance and T2DM. This is also supported by the link here between *BDNF* expression and circulating IGFBP-2, an important marker of insulin sensitivity [19].

It was demonstrated previously that BDNF affects glucose metabolism not only in central metabolic pathways but also in the peripheral glucagon secretion pathway [9]. Additionally, 3 weeks of BDNF administration significantly reduced blood glucose concentrations and glycated haemoglobin (HbA1c) in a db/db mice model [8]. BDNF not only temporarily reduced blood glucose concentrations but also ameliorated systemic glucose balance in this obese diabetic mouse model during the experimental period [8]. Given the positive association between higher circulating IGFBP-2 and greater insulin sensitivity in humans [19,20,21], the association between lower *BDNF* methylation (so potentially higher circulating BDNF) and higher circulating levels of IGFBP-2 is an important and relevant finding.

BDNF also plays an important role in fat accumulation in an animal model, since young rats under a hypercaloric diet showed an increase in adipose tissue concentration, and this was associated with reduced hippocampal *BDNF* expression [22]. Our results at baseline are in accordance with these findings, shown by the positive correlation we found between mean methylation in adipose tissue and waist/hip ratio, and the negative correlation between serum levels and waist/hip ratio, since if the methylation levels are high, we would expect lower *BDNF* expression accompanied by higher waist/hip ratio.

After the intervention, there was a reduction in *BDNF* mean methylation in buffy coat. Although we only observed a small number of individuals here, this finding suggests the idea that *BDNF* methylation may be influenced by dietary changes with consequent modulation of *BDNF* gene expression. We also found a correlation between the *BDNF* mean methylation in buffy coat and HbA1C levels. *BDNF* methylation is very poorly explored in the context of diabetes. So far, the DNA methylation of exon IV, a regulatory region of BDNF gene that contains a site for the transcription factor cAMP-response element binding protein (CREB), has been analysed by only one study [23]. CREB family transcription factors are required for the early induction of all the major BDNF transcripts [24]. The major findings were correlations with insulin levels and hip/waist ratio in specifics CpGs in newly diagnosed diabetes and pre-diabetes. Additionally, higher BDNF methylation was associated with high fasting insulin levels [23], supporting the hypothesis that BDNF methylation plays important role in diabetes progression.

In this study, we found that BDNF serum levels correlated with insulin post-intervention, and the delta values of BDNF serum and HOMA-S, an indicator for insulin sensitivity, were also inversely correlated. These findings reinforce the role of BDNF in glucose regulation [25,26] and the evidence showing negative interaction between impaired glucose tolerance and circulating BDNF levels [27], which could contribute to our understanding of the frequently seen association between diabetes and cognitive impairments [1,28].

Considering that epigenetic markers are modified by external factors, such as age, physical activity, in utero environment, and availability of nutrients, the transient and reversible nature of epigenetic modifications provides a vast field for discovery of targets for future prediction and therapeutic concepts in NDH and T2DM.

Our findings in this paper contribute to the knowledge about the epigenetic influence on the trajectory over time in relation to T2DM and add important evidence related to BDNF methylation supporting its role in glucose metabolism and in modification of eating behaviour. Future studies involving BDNF methylation, NDH, and T2DM patients are necessary to the development of new therapeutic targets and for future preventive, earlier interventions, such as more specific diets and tailored exercise regimes.

We accept that these findings are only based on a small number of people, and that there was no change in serum BDNF concentration with the lifestyle intervention. However, the group that we studied was fully characterised metabolically, and we were able to analyse BDNF methylation in both buffy coat samples and adipose tissue.

In conclusion, higher *BDNF* % methylation levels are known to reduce *BNDF* expression. The association between lower *BDNF* methylation (so potentially higher BDNF) and higher HbA1c/circulating IGFBP-2 (as a marker of insulin sensitivity) and between lower serum BDNF and higher circulating insulin suggests that the degree of *BDNF* methylation may be indirectly implicated in insulinisation and glucose homeostasis, particularly after lifestyle change in NDH individuals.

## 4. Materials and Methods

### 4.1. Samples

Serum, fat tissue, and whole blood were collected from 20 NDH individuals (10 male, 10 female) at baseline and following a 6-month telephone-led structured lifestyle intervention (Care Call programme) where they received exercise and nutritional advice with the aim of improving glycaemia and reducing weight. All 20 individuals participated in the Care Call programme. The Care Call programme is a modular telephone-based intervention programme utilising motivational support techniques, lifestyle education, and one-to-one and peer discussion and encouragement of progress with goals and signposting/referral to relevant services, with tailoring of content to individual needs (Supplementary File S1). The complete description of sample collecting and Care Call programme details were described previously by our group [17,18].

### 4.2. DNA Methylation

Genomic DNA was extracted from adipose tissue and buffy coat from all 20 individuals before and 6 months after the intervention using DNA Qiamp mini kit (Qiagen, Manchester, UK). Afterwards, the DNA was bisulfite converted using EpiTec Fast DNA Bisulphite Kit (Qiagen) to convert unmethylated cytosine residues to uracil with a calculated mean conversion of 99%. A pyrosequencing method was used for the determination of methylation at four CpG sites within the BDNF IV exon, as previously described by our group [29]. The sequence was amplified by PCR, using the primers described on Table 1. Pyrosequence setup and data reading were conducted by PyroMark Q48 using the version 2.4.2 software (Qiagen). The samples underwent PCR and pyrosequencing in duplicate; any inconsistencies between samples were resolved following further repetition.

### 4.3. Gene Expression

RNA was extracted from buffy coat using the RNeasy mini kit (Qiagen) and reverse-transcribed using the QuantiTect reverse transcription kit (Qiagen). The cDNA concentration and purity were determined using a NanoDrop Lite spectrophotometer (ThermoFisher Scientific, Waltham, MA, USA). Relative gene expression was determined using a QuantiStudio5 (Applied Biosystems, Waltham, MA, USA) and TaqMan^®^ reagents, with β-actin (ACTB) as housekeeping gene. The experiments were assayed in triplicate, and 50 ng of total cDNA was used per reaction for BDNF and ACTB. The PCR protocol was as follows: pre-incubation—1 cycle at 95 °C for 10 min, amplification—50 cycles at 95 °C for 10 s, 60 °C for 30 s, and 72 °C for 1 s, and cooling—1 cycle, 40 °C for 30 s.

### 4.4. Serum Protein and Other Assays

Serum quantitative determination of BDNF was performed by ELISA using the Human Free BDNF Quantikine ELISA Kit (R&D Systems, Minneapolis, MN, USA), according to the manufacturer’s instructions. The detection range was 62.5–4000 pg/mL, and the sensitivity was 20 pg/mL. Serum insulin-like growth factor binding protein-2 (IGFBP-2) concentration was determined by ELISA. The measurement of all other analytes reported here was described in previous papers [17,18].

### 4.5. Statistical Analysis

All analyses were carried out using the Statistical Package for Social Sciences (SPSS version 20.0, Armonk, NY, USA). Descriptive analyses were performed to evaluate socio-demographic and clinical characteristics and have been published elsewhere [17,18].

Gene expression was quantified using the comparative threshold (Ct) method (ΔΔCt method), and the amount of *BDNF* (target gene) was normalised to the housekeeping gene *ACTB* and determined by 2^−ΔΔCt^, with relative expression levels reported as fold change. Changes in *BDNF* gene expression, serum levels, and *BDNF* DNA methylation for each CpG or mean methylation before and after intervention were compared by paired *t*-test followed by Bonferroni correction for multiple comparisons as a post hoc test and were considered significant when *p* ≤ 0.025. Normality of distribution was assessed by the Kolmogorov–Smirnov test, and all the variables presented normal distribution (*p* > 0.05); thus, we performed parametric tests for our analysis. The BDNF serum levels, gene expression, and DNA methylation were then correlated with clinical cardiometabolic parameters by Pearson’s correlation considering r ≥ 0.35 and *p* ≤ 0.05 significant. As we did not see any effect of age and sex for any of the variables, pre and post intervention, we carried out our correlations without age and sex adjustment.

## Figures and Tables

**Table 1:** List of Forward (F) and biotinylated Reverse (R) primers used in PCR reactions, and Sequencing (Seq) primer for pyrosequencing.

	Primers
*BDNF*	F 5′GATTTTGGTAATTAGTGTATTAGAGTGTT3′ R 5′CCCCATCAACCAAAAACTCCATTTAATCTC3′ Seq 5′GGTAGAGGAGGTATTATATGATAG3′

## Data Availability

Request can be made to adrian.heald@manchester.ac.uk.

## Supplementary Materials

The following supporting information can be downloaded at: https://www.mdpi.com/article/10.3390/epigenomes6010007/s1, File S1. Care Call: Preventing type 2 diabetes.
